# The pharmacokinetic–pharmacodynamic modelling framework as a tool to predict drug resistance evolution

**DOI:** 10.1099/mic.0.001368

**Published:** 2023-07-31

**Authors:** Christopher Witzany, Jens Rolff, Roland R. Regoes, Claudia Igler

**Affiliations:** ^1^​ Institute of Integrative Biology, ETH Zurich, Zurich, Switzerland; ^2^​ Evolutionary Biology, Institute for Biology, Freie Universität Berlin, Berlin, Germany; ^3^​ School of Biological Sciences, University of Manchester, Manchester, UK

**Keywords:** antimicrobial resistance evolution, PKPD modelling, population genetics, dynamic selection pressure

## Abstract

Pharmacokinetic–pharmacodynamic (PKPD) models, which describe how drug concentrations change over time and how that affects pathogen growth, have proven highly valuable in designing optimal drug treatments aimed at bacterial eradication. However, the fast rise of antimicrobial resistance calls for increased focus on an additional treatment optimization criterion: avoidance of resistance evolution. We demonstrate here how coupling PKPD and population genetics models can be used to determine treatment regimens that minimize the potential for antimicrobial resistance evolution. Importantly, the resulting modelling framework enables the assessment of resistance evolution in response to dynamic selection pressures, including changes in antimicrobial concentration and the emergence of adaptive phenotypes. Using antibiotics and antimicrobial peptides as an example, we discuss the empirical evidence and intuition behind individual model parameters. We further suggest several extensions of this framework that allow a more comprehensive and realistic prediction of bacterial escape from antimicrobials through various phenotypic and genetic mechanisms.

## Introduction

Drug treatment of microbial infections is a key factor in alleviating disease morbidity, but we are fighting against organisms that can quickly evolve resistance to our drugs [1]. Hence, a crucial aspect in avoiding treatment failure is the appropriate use of antimicrobial drugs through treatment designs that maximize drug efficacy and minimize the potential for resistance evolution [2–5]. This effort is significantly aided and sped up by the use of mathematical modelling approaches, most notably pharmacokinetic–pharmacodynamic (PKPD) models [6–10].

PKPD models describe the time course of the drug concentration over the treatment and relate it to an effect on bacterial growth (Box 1). Despite their relative simplicity, PKPD models have been used successfully by the pharmaceutical industry as well as academia to predict bacterial killing efficacy for drug development and treatment optimization [6, 11]. However, in order to minimize antimicrobial resistance evolution, it is becoming increasingly important to consider not only killing efficacy but also the potential for genetic and phenotypic resistance evolution in PKPD models [6, 12–16].

Box 1. **Predicting resistance evolution during drug treatment by coupling pharmacokinetics (PK), pharmacodynamics (PD) and population genetics.**

PK models are used to describe the change in drug concentration over time. A single administration of a drug dose *A*_*adm*_ which is absorbed at rate *k*_*a*_ and eliminated (assuming first-order kinetics) at rate *k*_*e*_ can be modelled as [172]:

$$A(t)=\frac{k_{a}}{k_{a}-k_{e}}A_{adm}(e^{-k_{e}t}-e^{-k_{a}t})$$

If another drug dose is administered before the drug concentration is completely eliminated, the drug accumulates – potentially resulting in higher effective drug concentrations (*A*_*eff*_) than *A*_*adm*_ (see *A*_*eff*_ IV) – until drug input and drug elimination reach a steady state. An antimicrobial treatment regime consisting of *n* administrations of a drug dose *A_adm_* at intervals τ can be modelled by:

$$A(t)=\sum_{n}\frac{k_{a}}{k_{a}-k_{e}}A_{adm}(e^{-k_{e}(t-(n-1)\tau )}-e^{-k_{a}(t-(n-1)\tau )})H(t-(n-1)\tau )$$

where *H* is the Heaviside step function $H(t-(n-1)\tau ):=\left\{ \begin{array}{c} 0\;if\;t§lt;(n-1)\tau \\ 1\;if\;t\geq (n-1)\tau \end{array}\right.$ ,

which ensures that the new drug dose is only added after a period τ . Absorption and elimination – and therefore the drug kinetics – depend on the mode of administration, e.g. intravenous (IV, black line) or oral (blue line), and the drug type. Further, drug concentration changes and diffusion in the human body can be approximated more realistically by modelling two or more compartments [173].

The PD curve depicts the effect of the drug concentration on the net growth rate of bacteria. In the absence of drugs bacteria grow at a rate ψ_*max*_ and are killed with rate ψ_*min*_ at high drug levels $\left(\psi (A\gg MIC)=\psi_{min}\right)$ with ψ_*min*_<0 for bactericidal drugs, and ψ_*min*_=0 for bacteriostatic drugs. The difference between ψ_*max*_ and ψ_*min*_ represents the maximal drug effect (*E*_*max*_). The MIC shows the minimal drug dose that inhibits bacterial growth (ψ(A=MIC)=0) and the steepness of growth rate decrease with drug concentration is given by a Hill coefficient κ . The PD curve is therefore described by [26]:

$$\psi (A)=\psi_{max}-\gamma_{i}(A)$$

$$\gamma_{i}(A)=\frac{(\psi {}_{max}-\psi {}_{min}){\left(\frac{A}{MIC_{i}}\right)}^{\kappa }}{{\left(\frac{A}{MIC_{i}}\right)}^{\kappa }-\frac{\psi_{min}}{\psi_{max}}}$$

where γ*_i_* (*A*) is the death rate due to drugs for drug-susceptible (*i=S*) or drug-resistant bacteria (*i=R*) with *MIC*_*R*_ *>MIC*_*S*_. The benefit of a resistance mutation is given by the fold-increase of *MIC*_*R*_over *MIC*_*S*_. This drug-dependent growth rate of bacterial pathogens can then be incorporated into a population genetics model to predict population dynamics and resistance evolution [15]:

$$\frac{dS}{dt}=(1-u)\left(1-\frac{S+R}{K}\right)\psi_{max}\;S-\gamma_{S}(A)\;S$$

$$\frac{dR}{dt}=(1-c)\left(1-\frac{S+R}{K}\right)\psi_{max}\;R+u\;\left(1-\frac{S+R}{K}\right)\psi_{max}\;S-\gamma_{R}(A)\;R$$

Here ψ_*max*_ is the susceptible bacterial net growth rate, *c* the cost of resistance, *u* the mutation rate towards *R, K* the carrying capacity of the system, and γ_*S*_ and γ*_R_* the drug-induced death rates of the susceptible (*S*) and the resistant (*R*) population, respectively. Example parameter values for antibiotics and antimicrobial peptides are given in Table S1.

**Figure:**
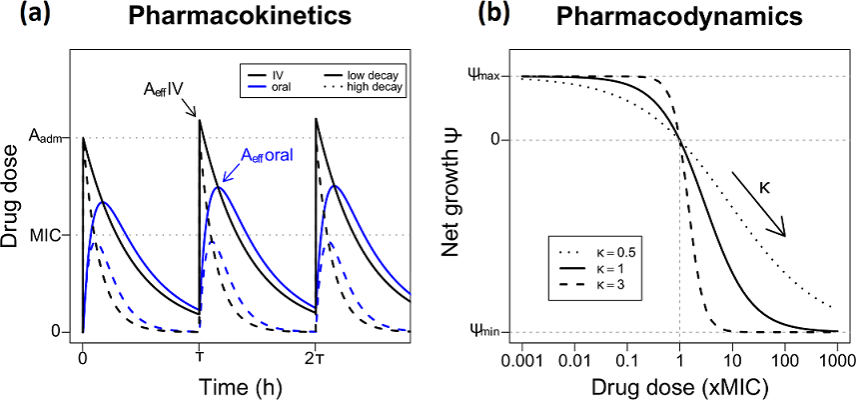

This review is intended as a primer on coupling PKPD and population genetics models and unlocking their power to predict and prevent drug resistance. The ultimate risk of resistance evolution results from the interplay of parameters describing the treatment regimen (PK model), bacterial susceptibility (PD model) and selection (population genetics model) [13]. We collate available empirical values for parameters of PKPD and population genetic models and discuss their effect on treatment outcomes. Further, we present extensions of this modelling framework that could enhance their predictive value. In particular, we exemplify how PKPD–population genetics modelling can be used to prevent the selection of drug resistance by comparing two antimicrobial drug classes, antibiotics (ABs) and antimicrobial peptides (AMPs).

## PKPD modelling

PKPD models are of direct relevance for clinical practice and drug approval by allowing the evaluation of: (1) *Pharmacokinetics*, which describe the concentration of drugs over time and throughout body compartments [6, 17, 18], and (2) *Pharmacodynamics*, which determine the effect of the drug concentration on the pathogen [6, 7, 19, 20] (Box 1).

## Pharmacokinetics (PK)

Optimizing the PK concentration profile is a crucial prerequisite for drug treatment success and can be adjusted via dosing, timing and route of drug administration. The initial drug dose administered to a patient (*A_adm_*) is dictated by the drug susceptibility of the pathogen population, and limited by toxicity considerations [21]. However, depending on the drug type and administration route, the effective dose (*A_eff_*) at the infection site can differ substantially from the applied dose due to delayed distribution or conversion between drug forms [6, 22, 23]. With oral administration for example, *A_eff_* is probably lower than *A_adm_* as the drug declines exponentially with rate *k_e_* due to drug elimination through metabolism and excretion, while it diffuses to the target sites. Therefore, to achieve sufficiently long periods of *A_eff_* exceeding the MIC, drugs are often administered multiple times at frequency τ, which is determined by the drug half-life $\left(t_{\frac{1}{2}}=\frac{ln\left(2\right)}{k_{e}}\right)$ . The overall treatment duration (*t_max_*) is equal to the number of drug administrations (*n*) times their frequency (*t*_*max*_=*n*) and is chosen to maximize pathogen eradication. As only a few PKPD models have as yet taken into account the host immune system [24], the necessary treatment duration calculated with PK models is probably a conservative estimate.

## Pharmacodynamics (PD)

While PK models can capture the kinetics of drug concentration, their stand-alone usefulness in predicting pathogen dynamics is limited – as bacterial killing is not linearly related to drug concentration [25, 26]. Therefore, PD models are used complementarily to PKs to describe the sigmoidal relationship between (effective) drug concentration on the net growth rate of the pathogen population. The best-known PD parameter is the MIC, which describes the lowest drug concentration that stops pathogen growth, and is used as a standardized clinical measure to assess susceptibility of a given pathogen to a specific drug [27]. Drug treatment regimens are often based on this value alone, and hence it is available for a wide range of antimicrobials and bacterial species. Despite its ease of application, the MIC has several shortcomings, from a general paucity of accuracy [28] to its limited correlation with drug efficacy [26] and tolerance [29].

The MIC is however only a single point on the PD curve (see Box 1), which more generally describes how much a species’ net growth rate *ψ*(*A*) is reduced with increasing drug concentration from its maximal growth rate *ψ*_*max*_ (i.e. in the absence of drugs). Despite potential implications for antimicrobial efficacy [30], variations in *ψ*_*max*_ within a pathogen population are usually not considered in PKPD models. The effect of increasing drug concentration on bacterial growth levels off when *ψ*_*min*_, the minimal net growth rate or maximal killing rate, is reached (i.e. *ψ*_*min*_≤0). This parameter reflects the maximal speed of pathogen eradication. Note that time-kill curves via OD measurements do not allow for quantification of *ψ*_*min*_<0. Studies using plating assays, however, showed that *ψ*_*min*_ can vary drastically between antimicrobials [26, 31, 32]. The steepness of the sigmoidal PD curve connecting *ψ*_*max*_, *MIC* and *ψ*_*min*_ is given by κ (Box 1), which describes the sensitivity of the bacterial population growth rate to an increase in antimicrobial concentration. The estimated value of κ is dependent on the estimated value of *ψ*_*min*_, meaning that the estimates can differ between studies using OD measurements and plating assays for growth determination.

## Population genetics modelling of resistance evolution

While PKPD models can accurately capture the strength of selection pressure as determined by the antimicrobial concentration over time, population genetic models translate this selection pressure to changes in genetic composition within a population. In the context of antimicrobial resistance, the relevant genetic variation is linked to the susceptibility to the antimicrobial. In population genetic models, these changes in susceptibility are generally considered to emerge via mutations, which are then either selected for and rise to fixation or die out, depending on the selection pressure and stochastic effects. Mutation rates are hence one of the most important factors in determining the speed of resistance emergence in these models. While the overall genomic mutation rate depends largely on the pathogen species, the effective resistance mutation rate depends on the number of possible mutations that can confer resistance and hence on the drug type [33] as well as environmental factors [34–37].

Even though the emergence of a resistant sub-population is heavily influenced by the mutation rate, the fixation of a resistance mutation is determined by subsequent selection, which is described by PKs and PDs (Box 1). Hence, coupling bacterial population genetic models with PKPD models allows for the study of both,treatment efficacy as well as resistance evolution under various drug treatment regimens [6]. Notably, population genetic models can also be modified to study resistance caused by horizontal gene transfer [13, 38] or phenotypic resistance [14, 30].

## Selection pressure and the mutant selection window

A suboptimal PK concentration profile can not only hinder treatment success, but potentially even facilitate resistance emergence by selecting resistant subpopulations. For example, prolonged periods of subinhibitory drug doses can speed up the emergence and spread of resistance by increasing heterogeneity in the pathogen population and eliminating susceptible competitor strains [39–41]. Subinhibitory drug regimens could, however, in principle slow down *de novo* resistance emergence by increasing stochastic loss of rare resistant cells [42]. Generally, subinhibitory drug levels should be avoided by adjusting the frequency of drug administration according to the half-life of a drug [43]. Drugs with very long half-life in humans, such as dalbamycin (=6–11 days [44]), do therefore not require frequent dosing to stay above the MIC and facilitate out-patient treatment [45]. On the other hand, drugs with short half-lives require careful consideration of the speed of drug diffusion to the target site and conversion into an active form to properly gauge the effective PK and adjust dosing frequency accordingly [46]. However, drugs with short half-lives have the potential to minimize subinhibitory drug periods in patients after treatment ends, thereby reducing selection pressure for environmental persistence of resistance [47].

Accordingly, setting the overall treatment duration (*t_max_*) poses a tradeoff between eradicating the pathogen population, considerations of toxicity and resistance selection. Typical recommendations for antibiotic treatments vary from days to weeks [48], according to the pathogen. The longer the treatment, the more crucial is the use of optimal drug regimens [49, 50] as selection for *de novo* resistance or horizontal gene transfer (HGT) of pre-existing resistance from the non-pathogenic host microbiota increases.

**Fig. 1.**
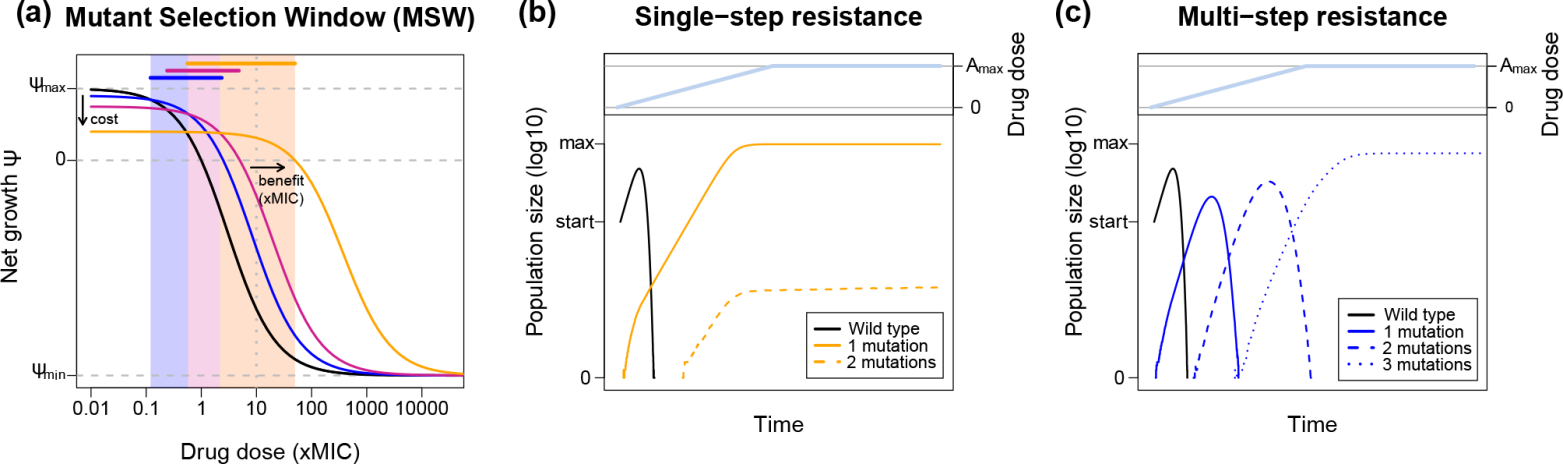
Predicting the dynamics of genetic resistance evolution. (**a**) Comparison of the pharmacodynamic dose–response curves of susceptible (black line) and increasingly resistant mutants (blue, pink and orange lines). Resistance is here characterized as a mutation that provides a higher MIC than for the susceptible bacteria (MIC_R_ >MIC_S_) and a reduction in maximal growth rate due to cost of resistance (*ψ*_*max,R*_<*ψ*_*max,S*_). Mutations with higher resistance benefits, i.e. higher MIC increases, are usually accompanied by higher costs. The drug dose range, where resistant mutants have higher (non-zero) net growth rates than the susceptible bacteria, selects for resistance and is therefore called the mutant selection window (MSW). If each of the three shown mutations arises in a wild-type population, the size of the MSW depends on the relative benefit and cost of a resistance mutation compared to the wild-type (represented by coloured bars). If multiple mutations are present simultaneously, the size of the MSWs depends on the comparative benefits and costs of those mutations in relation to all other genotypes in the populations, which generally shrinks the MSW (represented by shaded areas). Mutations that provide a high MIC increase are likely to provide resistance with a single mutation and additional mutations are not selected for (**b**), whereas mutations with a small MIC increase are likely to lead to sequential acquisition of multiple mutations with additive or multiplicative effects (**c**). See Text S1, available in the online version of this article, for details on the modelling framework underlying (**b**) and (**c**).

The enrichment of resistant mutants over the course of treatment is determined by the differences in the effect of antimicrobial concentration on the growth of susceptible and resistant bacteria. Essentially, resistant mutants are selected for at antimicrobial concentrations where their net growth rate is higher than the net growth rate of the susceptibles and greater than zero (Fig. 1). This concentration range is called the mutant selection window (MSW). The lower limit of the MSW is given by the antimicrobial concentration at which resistant and susceptibles have equal growth rates – the minimal selective concentration (MSC) – and the upper limit by the MIC of the resistant mutant MIC_R_.

Hence, the lower limit of the MSW is affected by any fitness costs caused by a resistance mutation, i.e. growth reductions in drug-free environments. Such reductions in *ψ*_*max*_ will make the MSW smaller and thereby decrease the establishment potential of mutant cells. Fitness costs can also decrease pathogen virulence, but many studies show unaffected or even increased virulence [51]. While reversion of resistance mutations is rare [52, 53], fitness costs of resistance can be mitigated by compensatory mutations – though often only to a certain extent as compensatory mutations can come with their own costs [54].

The maximal killing rate *ψ*_*min*_ does not influence the size of the MSW directly, but it reflects the time until the pathogen population is eradicated and hence the timespan in which resistance can potentially arise. Values of *ψ*_*min*_ are rarely reported in different environments and can vary with cell and resource density [11], but might not be strongly affected by resistance mutations [55]. The risk of resistance evolution could, however, depend on the mode of action, i.e. bacteriostatic (*ψ*_*min*_) or bactericidal (*ψ*_*min*_<0) [21].

Overall, while an increase in MIC indicates resistance (MIC_R_>MIC), it does not necessarily translate to treatment failure, depending on the drug concentration and killing dynamics. Importantly, while antimicrobial concentrations within the MSW will select for resistance, it is necessary to assess all PD parameters, including *ψ*_*max*_, *ψ*_*min*_ and κ, of resistant and susceptible bacteria, to understand the evolutionary dynamics of resistance emergence. For example, large values of κ, describing steep PD curves, result in a narrow MSW, while small values of κ result in a wide MSW. Intuitively, large values for κ reflect that sub-MICs have little effect on bacterial growth, while only an incremental concentration increase from the MIC is necessary to reduce growth to *ψ*_*min*_ [15, 31].

## PKPD modelling explains differences in resistance evolution against ABs and AMPs

AMPs and ABs represent two major and extensively studied classes of antimicrobials with high clinical significance [56, 57]. The distinct structural and functional characteristics of AMPs and ABs [58, 59] translate to consequential differences in PK and PD parameters (Table S1). Hence, we will use them as a compelling example to illustrate how differences in PK and PD parameters can be used to predict drug efficacy, the emergence of resistance and the optimality of treatment design.

AMPs are antimicrobials that are ubiquitously found as part of plant and animal defence systems against bacteria, viruses and fungi [58] and because of their diversity are considered as promising candidates for drug development. AMPs are peptides with spatially explicit hydrophobic and cationic residues, providing them with a much higher affinity for negatively charged prokaryotic membranes than eukaryotic membranes [60]. Hence, AMPs are typically active from the outside, whereas ABs are usually molecules that target a variety of essential structures within the cell. This difference in targets is reflected in (effective) resistance mutation rates, which are generally lower for AMP resistance than for AB resistance [61] (Table S1). Further, as opposed to several ABs [35, 62], AMPs do not induce mutagenesis via the stress response [61], although they might still promote mutagenesis under specific circumstances (such as interaction with free iron) [63].

## Optimizing PK curves is more challenging with AMPs

Peptides, such as AMPs, are typically subject to proteolytic activity, which can result in significantly lower effective concentrations than the administered one. For instance, orally administered AMPs are likely to be degraded before absorption in the gut (e.g. *A_eff_* =0.4–15 % of *A_adm_* [64]). ABs, on the other hand, are relatively stable with half-lives of several hours [43, 65] (Table S1). Hence, the same route of drug administration can result in substantially different PKs for ABs and AMPs. Further, AMP absorption is gradual and hence peak drug levels are delayed, which seems to mimic their natural production rates [66]. Surprisingly, the potential for resistance evolution against AMPs was found to be higher for such delayed PKs [13], while for ABs the potential for resistance seems to be less sensitive to the speed of absorption [13]. This suggests that the current practice of frequently administering AMPs intravenously [67] and ABs orally could already work towards minimizing resistance evolution.

In addition to the route of administration, sub-optimal PK profiles can be compensated for by increasing the frequency of administration or dosage as far as toxicity considerations allow. While toxicity has been a constraint only for a minority of ABs (e.g. fluoroquinolones [68]), potential toxicity for host tissues has been a main concern with AMPs [33, 69]. However, to date, several natural [70–72] and synthetically engineered [73, 74] AMPs with negligible cytotoxicity have been discovered. Side effects of antimicrobial drugs are also related to their specificity for the pathogen, as opposed to our essential microbiota. As ABs are effective against a broad range of bacteria, they generally can have long-lasting effects on the host gut microbiota [75] and potentially even on the host immune system [76]. For some AMPs, on the other hand, recent studies suggest an unexpected specificity [57], which could aid in reducing unintended side-effects.

## PD features reveal lower resistance potential against AMPs

For ABs, PD indices based solely on the MIC have been developed to predict bacterial killing [77] and resistance evolution [78] in clinical use. In some cases these predictions were successful [43, 79–81], but other studies did not find a consistent correlation with the chosen PD index [65, 82, 83], potentially because predictions based solely on the MIC neglect more intricate interaction dynamics between bacteria and antibiotics [80, 84, 85]. However, individual PD parameters can still be useful to gain an intuition about the potential for resistance evolution against different types of antimicrobials [15]. For example, compared to ABs, AMPs have much lower *ψ*_*min*_ values, allowing AMPs to kill cells within minutes [32, 86–89] (Table S1), which lowers the potential for resistance evolution [15]. The killing speed of ABs on the other hand seems to be on the order of hours [26, 90, 91] (Table S1), probably because their mechanism of action requires entering bacterial cells. Further, while ABs show a wide range of κ values [26, 92], AMPs tend to have mostly high κ values (Table S1), which (together with a lower *ψ*_*min*_) leads to a smaller MSW and therefore reduces resistance evolution against AMPs [15]. In contrast to what has been observed with some ABs [92], however, the κ of AMPs can be affected by resistance mutations [55].

The evolution of κ illustrates that potentially other PD parameters, not just the MIC, are affected by resistance evolution [93]. For instance, resistance usually decreases *ψ*_*min*_, the growth rate in the absence of antimicrobials, which decreases the size of the MSW. Reported fitness costs of single resistance mutations against ABs [70, 92, 94] and AMPs [33, 70, 95–99] vary over a wide range (including no costs [100–102]), but are on average within the same order of magnitude (Table S1). As mutational benefits (e.g. MIC increases) are generally lower with AMPs than with ABs [103] (Table S1), this indicates on average higher costs relative to the benefit, which slows resistance emergence. This is further corroborated by the fact that, while ABs show facilitation of drug resistance evolution at sub-MIC drug levels [34–36], no such facilitation has been found with AMPs so far [104].

Overall, PKPD models can be very useful in distinguishing the optimality of different treatments to achieve treatment success and resistance avoidance.

## Modelling framework extensions

Coupled PKPD–population genetics models can be very powerful tools in understanding bacterial adaptation, not only via mutations, but also via HGT and phenotypic changes. Yet, this framework is rarely used to this end and in the following we will suggest extensions that could be used to explore bacterial adaptation more comprehensively.

## Distribution of fitness effects (DFEs)

If modelling studies include resistance evolution, they usually consider the emergence and spread of a single mutation providing ‘full’ resistance, i.e. higher than the applied drug dose [6]. However, in reality, mutational resistance provides a fold increase in drug MIC and can span orders of magnitude [13]. The magnitude of the mutational benefit is potentially linked to the resistance mechanism [105]: higher benefits are often conferred through specific drug target mutations, whereas lower benefits occur with unspecific mechanisms such as efflux pumps. In agreement with this, mutational benefits for AMP resistance – which frequently arises from unspecific membrane modifications [97, 99] – seem to be generally smaller than for AB resistance, which often targets specific cellular functions [70, 92, 94, 97, 99, 106]. The number of mutations that is necessary for full resistance will therefore depend on their benefit and the applied drug dose (Fig. 1, see Text S1 for details). Resistance could evolve either via a single mutation of a large benefit or via multiple mutations that each confer a small benefit. Across various ABs, both single- and multi-step evolution have been observed [34, 107–111], whereas AMP resistance mainly evolves via multiple mutational steps [70, 109, 112, 113], potentially slowing down AMP resistance evolution [13].

An important quantitative tool for predicting resistance evolution pathways are therefore distribution of fitness effects (DFEs). DFEs describe the proportion of mutations that will be beneficial, deleterious or neutral for a certain population in a given environment. Hence, they indicate the likelihood and effect (e.g. fold increase in MIC) of adaptation. While DFEs have been characterized for beneficial mutations in general [114], experimental determinations of drug resistance mutational ranges are rare. Moreover, DFEs are probably environment-dependent [115]; for example the width of the distribution was found to change in the presence of ABs [92]. In-depth determination of DFEs in the presence of ABs and AMPs will be crucial in modelling the dynamics of resistance evolution, particularly for resistance via multiple mutations.

## Horizontal gene transfer (HGT)

So far, the PKPD models that consider resistance evolution have mainly focused on mutational emergence, but resistance can also be acquired and sustained via HGT [38]. HGT promotes between-species resistance transfer [116] and often provides multi-drug resistance [117]. Resistance evolution via HGT could be integrated into the PKPD–population genetics framework as a transfer parameter, describing a certain uptake rate from the environment and from other resistant bacteria (e.g. the microbiota). For ABs, uptake rates are likely to be high as plasmid-related resistance is abundant [116, 118, 119]. For AMPs, a study of the human gut microbiota showed significantly less prevalence and spread of plasmid-related resistance [120] (even though our microbiota harbours resistance to many endogenous AMPs). Further, the potential for resistance being conferred through random short genomic fragments is low for AMPs but high for ABs [70, 120]. This trend is reflected in the soil microbiome being a large reservoir for AB [121], but not for AMP [70], resistance genes. Hence, the model parameter for HGT-acquired resistance should be lower for AMPs.

## Antimicrobial combination treatments

Although fixed combination formulas remain rare, empirical combinations of antimicrobials are an attractive clinical practice to combat resistance and increase treatment efficacy [122]. The difficulty with these approaches lies in predicting (and even defining [123]) the synergistic effects of multiple drugs, and in pre-empting cross-resistance to multiple antimicrobials. PKPD models have been successfully used to capture bacterial killing by incorporating empirically motivated drug interaction functions [124, 125] and have the potential to predict resistance evolution against drug combinations as well.

The aim of using two or more antimicrobials is to obtain a combined ‘effect’ (killing efficacy and/or resistance avoidance) that is superior to that of either drug alone – and ideally even superior to the sum of individual effects, i.e. a synergistic drug interaction. Between AMPs synergies are common in pathogen killing [31, 126] and they can also constrain resistance evolution [101, 127] – which could explain their natural occurrence in cocktails [128]. The beneficial effect for resistance avoidance from combining AMPs is caused by a steeper κ and, hence, a narrower MSW [31]. Often, the most effective killing efficacy is not achieved at maximum peptide concentrations [126], indicating functional interactions between AMPs. Synergies have also been found between AMPs and ABs [57, 71, 129–131], with AMPs possibly ‘opening the (membrane) door’ for intracellularly functional ABs [132], potentially re-sensitizing resistant pathogens [57]. For ABs killing synergism [133] and antagonism [134, 135] are both frequent, which can be understood at a mechanistic level when considering the cellular targets [136] and the AB-related growth inhibition of specific cellular functions [137]. Changes in κ for AB combination studies have shown no clear trend so far [138]. Mechanistic knowledge about drug actions can be used in PKPD models to design realistic interaction functions [124].

Worryingly, synergistic killing efficacy in drug combinations can lead to stronger selection pressure and therefore to faster AB resistance evolution [2–5]. This highlights the importance of combining drugs with a low risk for cross-resistance, meaning that a single resistance mutation provides protection against several drugs. Cross-resistance commonly arises between drugs with similar cellular targets, as exemplified by frequent cross-resistance between AMPs [97, 98, 128] and sometimes between AMPs and ABs [98, 139]. Congruently, an AMP that acts intracellularly did not show cross-resistance with other AMPs or ABs [100]. AMP cross-resistance is particularly concerning as it could decrease pathogen sensitivity to the AMPs of our immune response [33, 96, 99]. AB cross-resistance with at least one other drug is also relatively common (for more than 50 % of drug pairs tested [140]). However, AB-resistant bacteria rarely show cross-resistance with AMPs, but rather cross-sensitivity [141], which decreases the potential for multi-drug resistance. Understanding these patterns of cross-resistance and cross-sensitivity better and incorporating them in PKPD models will be essential for efficient design of antimicrobial combination treatments.

## Phenotypic resistance

**Fig. 2.**
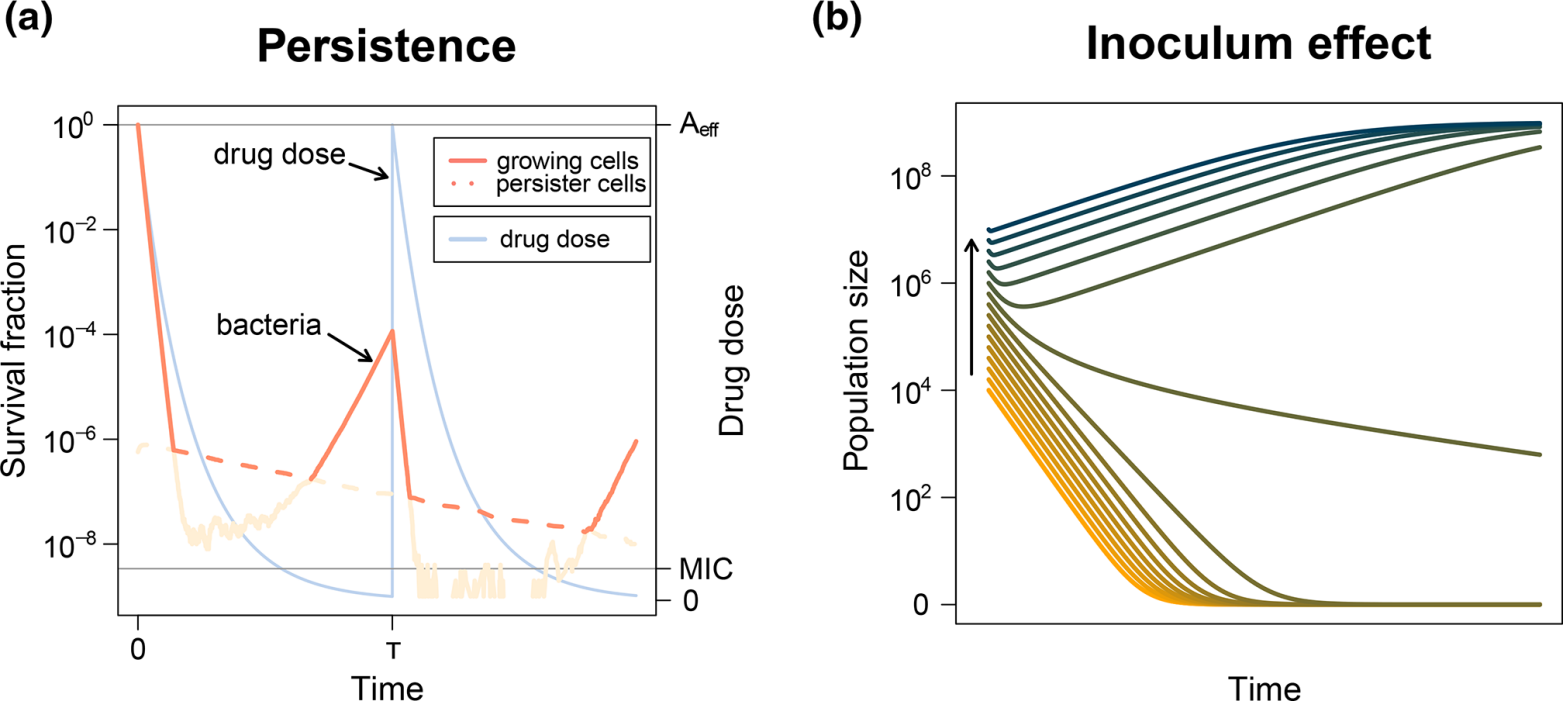
Predicting the dynamics of phenotypic resistance. (**a**) Bacterial persistence is characterized by the formation of a sub-population of transiently slow- or non-growing persister cells, which are recalcitrant to antibiotics. This gives rise to two distinct killing phases: rapid eradication of the larger, growing subpopulation followed by a slower decline of the persister subpopulation. Persisters die when they switch back to the growing state in the presence of antibiotics at >MIC. However, if the concentration is <MIC they can start growing and allow the population to survive treatment. Growing cells are shown by solid lines, whereas persister cells are shown by dashed lines. The currently dominating subpopulation is highlighted in salmon, whereas light-coloured lines indicate the ‘hidden’ minority subpopulation over the course of the treatment. (**b**) The inoculum effect describes the phenomenon that the MIC and the time until eradication increase with the initial population size (inoculum). This can, for example, be explained intuitively by uptake of antibiotic molecules by the cells. For details on the integration of persistence and inoculum effect into the PKPD framework see Text S2 and S3, respectively.

Although not genetically inheritable, phenotypic resistance [29, 142], which often arises from phenotypic heterogeneity within bacterial populations [30], can diminish drug efficacy and facilitate genetic resistance evolution [143–145]. Phenotypic heterogeneity is rarely quantified in resistance evolution experiments, but plays an important role in bacterial survival during drug treatment, as exemplified by slow- or non-growing persister subpopulations [146–148]. Incorporating persister dynamics into the PKPD–population genetics framework (Fig. 2a, see Text S2 for details) allows us to assess their impact on clearance and relapse of infection as well as the probability of resistance evolution [14, 149–151].

The mode of action of an antimicrobial can determine its effectiveness against persisters, depending on how much metabolic activity is necessary for antimicrobial function. Intracellular ABs such as ciprofloxacin and linezolid, for example, show very limited success, while it was found that the AMPs WR12 and D-IK8 can completely eradicate persisters [71]. This is likely because AMPs work extracellularly and do not require metabolically active cells. Exposure to antimicrobials, also called priming, might even be counterproductive and contribute to the formation of persisters, as was shown with ABs [151, 152] as well as AMPs [149].

Similarly, their activity at the surface of bacterial cells is probably the reason that AMPs have been successfully used against biofilms [131, 153, 154]. Biofilms are complex and potentially diverse structures that consist of bacterial cells enclosed in an extracellular matrix of polysaccharide and are often formed at infection sites. Biofilms are typically less amenable to AB treatment due to limited penetration of antimicrobial molecules and low metabolic activity of many bacteria in such biofilms [71, 142, 155, 156]. The PKPD framework coupled with population genetic approaches has recently also been successfully applied to biofilms [157, 158]. A quite surprising finding of these studies is that, while biofilms allow bacteria to evolve resistance at higher drug doses than for planktonic cells, they also impede resistance evolution at drug concentrations at which planktonic cells will evolve resistance.

Furthermore, the susceptibility of a pathogen population to antimicrobials can depend on the number of bacterial cells [159–161]. For both ABs [162] and AMPs [89, 159, 163–165], the efficacy of an antimicrobial decreases with increasing bacterial starting inoculum (called the ‘inoculum effect’), meaning that a higher number of bacterial cells increases the probability of survival and the potential for resistance evolution (Fig. 2b, see Text S3 for details). The inoculum effect can, for example, be caused by the uptake of AB molecules into bacterial cells, which depletes them extracellularly [159]. Quantifying the exact amount of AB molecules needed to kill a single cell is difficult as the binding of intracellular targets leads to a gradual increase in the probability of cell death [159, 166, 167]. For AMPs, killing thresholds are more defined, with millions of molecules needed to bind to the membrane of a single cell to disrupt it [86, 164]. Further, up to 10 million AMP molecules can bind to targets made accessible inside each dead and permeabilized cell [168, 169]. These different killing dynamics might give rise to different dependencies on bacterial densities for ABs and AMPs.

Surprisingly, bacterial density can also influence mutation rates, generally decreasing them at higher cell numbers [37]. Hence, resistance evolution might be slower at high cell numbers, but antimicrobials are also less effective at killing bacterial cells. The PKPD framework can help to isolate the contributions of density-dependent effects on clearance and resistance emergence for antimicrobial treatment regimens, which may be difficult to discern experimentally.

## Concluding remarks

It is the combination of resistance emergence and selection for the resistance that endangers the success of antibiotic treatments [170]. Coupling PKPD and population genetics models is a uniquely powerful tool to assess these two processes in clinically relevant environments: PK curves of antimicrobials describe the fluctuations in the pathogen environment over time that the PD curve translates into a selection pressure acting on a pathogen population, whose adaptation is described by population genetics. Crucially, these models consider that pathogen fitness, and therefore selection pressure, varies over time in dependence of the antimicrobial concentration [171], but also due to adaptive changes in the pathogen population. Another strength of the PKPD–population genetics approach is that it builds on individually well-studied components (e.g. the spread of mutations in bacterial populations or the distribution of antimicrobials throughout the human body), to give a comprehensive picture of pathogen dynamics.

By using ABs and AMPs as an example, we show here how various model parameters intuitively provide an understanding of drug efficacy and the risk of resistance – even though their interplay remains complex and necessitates evaluation of the full model to predict bacterial adaptation under drug treatment. This effort will also require more systematic empirical quantification of several, currently under-reported, parameters (e.g. κ and DFEs) to allow a better reflection of clinical reality in mathematical models [170].

## Supplementary Data

Supplementary material 1
