# Supplementary material for: The pharmacokinetic–pharmacodynamic modelling framework as a tool to predict drug resistance evolution
Source: Microbiology (Reading). 2023 Jul 31;169(7):001368. doi: 10.1099/mic.0.001368 (PMC10433423; doi:10.1099/mic.0.001368)
Supplement: Supplementary material 1 [file mic-169-1368-s001.pdf]

Supplemental Information for

The pharmacokinetic-pharmacodynamic modeling framework as a tool to predict drug resistance evolution

Christopher Witzany[1], Roland Regoes[1], Jens Rolff[2], Claudia Igler[1, 3,*]

[1] Institute of Integrative Biology, ETH Zurich, Zurich, Switzerland

[2]Evolutionary Biology, Institute for Biology, Freie Universität Berlin, Berlin, Germany

[3] School of Biological Sciences, University of Manchester, Manchester, UK

[*] Corresponding author: Claudia Igler (claudia.igler@manchester.ac.uk)

Text S1. Modelling resistance mutations with different effect sizes. The costs (i.e., growth reduction) and benefits (i.e., xMIC increase) of resistance mutations influence whether resistance against treatment emerges via a single or multiple mutational steps. We illustrate this by modelling resistance evolution against drug treatment with ramp PKs, i.e., drug dose increases linearly over time until a maximum dose A_{max} is reached which stays constant for the rest of the treatment. We simulate two scenarios: 1) a single mutation is sufficient to increase the MIC above A_{max} and 2) multiple sequential mutations are necessary to reach an MIC above A_{max}. Each of the mutations is accompanied by a cost that is proportional to its benefit (i.e., MIC increase). Note that in both scenarios up to three sequential mutations can arise but their selection depends on the combination of drug concentration and mutational effect. The stochastic simulations are implemented in R (version 4.2.0) using the open-source package *multistep.resistance* developed in[1].

Text S2. Modelling the dynamics of bacterial persistence. Bacterial persistence is caused by a phenotypic switch that can be described by an ODE model with two subpopulations[2–4]:

$$\frac{dG}{dt} = (\psi(A(t)) - s_F)G + s_B P$$

$$\frac{dP}{dt} = s_F G - s_B P$$

The growing subpopulation (G) is affected by the drug treatment according to the pharmacodynamic function $\psi(A(t))$, which describes the effect of the drug dose at a given time $A(t)$ on the bacterial net growth rate (see Box 1 in the main text for details). The phenotypically non-growing persister subpopulation (P) is refractory to drugs. Cells switch stochastically back and forth between the growing and persister state according to the rates s_B and s_F. Switching back to a growing state while the drug concentration is above MIC leads to cell death. Note, that this model describes spontaneous persistence (also known as type II persistence), i.e., switching rates are constant over time and independent of the environment[5]. We use the switching rates for wildtype *Escherichia coli* reported by Balaban et al., (2004) (see Table S1). The described stochastic population model is implemented in R (version 4.2.0) using the *adaptivetau*[6,7] package.

Text S3. Modelling the dynamics of the inoculum effect. The Inoculum Effect (IE) describes the phenomenon that with increasing population size (inoculum) the minimal concentration to inhibit growth (MIC) increases[8]. Multiple mechanisms can contribute to the IE, ranging from intrinsic drug-target binding kinetics[9,10] to extracellular enzymatic degradation of drug molecules[11]. A simple and intuitive form of IE arises when drug molecules get used up upon killing a single cell. Effectively, this is what happens for antimicrobial peptides (AMPs): after AMPs kill the bacterial cell by disrupting the membrane, AMP molecules in the vicinity of the cell can quickly and irreversibly be sequestered by intracellular targets inside the dead cell[12–15]. Such instantaneous off-target sequestration of drug molecules by dead cells can be captured by PKDP models[14]:

$$\frac{dB}{dt} = \psi_{max} B \left(1 - \frac{B}{K}\right) - \gamma(A)B$$
$$\frac{dA}{dt} = -N\gamma(A)B$$

The bacterial population (B) grows logistically with rate ψ_{max} and is limited by the carrying capacity K. The killing-effect of the drug (A) is described by $\gamma(A)$ according to the pharmacodynamic function (see Box 1 in the main text for details). The drug dose A decreases for every bacterium killed ($-\gamma(A)B$) by immediate uptake of N drug molecules.

Table S1. PKPD and population genetic model parameter values

Model parameter	Estimates
Drug bioavailability, A_{eff}	AB[16,17]: IV: 90 – 100% of A_{adm}; oral: 30 – 100% of A_{adm}; intramuscular: 84 – 88% of A_{adm}<br>AMPs: 0.4-15% of A_{adm}[18]
Drug absorption (passive diffusion), k_a	AB: 0.708 – 0. 899h^{-1} [19]; 1.074h^{-1} [20]
Drug half-lives, $t_{\frac{1}{2}} = \frac{ln(2)}{k_e}$	ABs: 4 – 12h[21,22]; 0.7 – 7.8h[17]<br>AMPs: 1 – 2h[23]
Maximal growth rate, ψ_{max}	*In vitro* measurements: 0.7 – 0.88h^{-1} [24]; 1.8h^{-1} [25]<br>*In vivo* measurements: -0.1 – 0.67h^{-1} [26]; 0.21 – 0.42h^{-1};
Minimal growth rate (maximal killing rate), ψ_{min}	AB: -8.8 – -4h^{-1} [24]; -2.3h^{-1} [25]<br>AMP: -50h^{-1} [27]
Sensitivity of drug response, κ	AB: 0.61 – 9.1, mean ~1.5[24,28]<br>AMP: 2.5 – 7.5, mean ~5[29]
Speed of action (related to ψ_{min} and κ)	ABs: 45min[30], 6h[31], 20 – 40min[24]<br>AMPs: 40sec – 4min[32]; 5-10min[33,34]; <2min[35]; 15 – 90min[36]
Drug diffusion through inner and outer bacterial cell membrane (related to speed of action)	AB: 2×10^{-11} m/s[9]<br>AMPs: NA (mostly active from the outside)
Mutational benefit, *b*	AB: (geometric) mean benefit: 28.0 ($\pm$ 7.1) xMIC/mutation<br>AMP: (geometric) mean benefit: 4.5 ($\pm$ 5.3) xMIC/mutation[1]
Fitness cost, *c*	AB: mean cost: 0.10 ($\pm$ 0.07) xMIC /mutation<br>AMP: mean cost: 0.04 ($\pm$ 0.03) xMIC/mutation[1]
Mutation rate, μ	General spontaneous mutation rate: 2.2×10^{-10} /nucleotide/gen; 10^{-3} /genome/gen [37]<br>ABs: 2×10^{-5} – 10^{-9}/cell/gen[38]; 4×10^{-9} – 10^{-8}/cell/gen [39]; 1.5×10^{-8} /cell/gen[40]; mutation frequency: 8.7×10^{-8} – 10^{-9}; mutators: 9.4×10^{-6} – 10^{-8} [41]<br>AMPs: 0.4×10^{-6} /cell /generation[42]; 0.4×10^{-6} – 2.3×10^{-7}/cell/gen[38]; 1-4× 10^{-9}/cell/gen [39]

Pathogen counts at infection sites (for estimating initial numbers and carrying capacity, *K*)	$2\times 10 – 4\times 10^{9}$ [43]; $4.5\times 10^{3} – 3\times 10^{8}$ [44], $10^{3} – 2\times 10^{9}$ [45], $5.3x\ 10^{3}$ – $1.8x\ 10^{11}$ [46] pathogen cells/ml
Persistence switching rates<br>(Type II / stochastic persistence)	To persister state:<br>Wildtype: $1.2\pm 0.2\times 10^{-6}\ h^{-1}$ [2]; $0.022\ h^{-1}$ [47]; $4.8\times 10^{-4}\ h^{-1}$ [48]; 0.21×10^{-3} and $1.2\times 10^{-3}\ h^{-1}$ [49]<br>High-persistence mutants: $1.0\pm 0.2\times 10^{-3}$ [2]; $0.33 – 0.62\ h^{-1}$ [48]<br><br>Out of persister state:<br>Wildtype: $0.1\pm 0.05\ h^{-1}$ [2]; 2.2×10^{-14} [47]; $1.26\ h^{-1}$ and $2.26\ h^{-1}$ [49]<br>Highpersistence mutants: $10^{-7} – 10^{-4}$ [2]<br>Primed: 0.28 and $0.40\ h^{-1}$ [49]
Sequestration rates of antimicrobials by dead cells (Inoculum effect)	AMPs:<br>LL37: 2.7 – 70 (µg/ml) $\times\ min^{-1} \times$ (dead cell/nl)$^{-1}$ [12]; 3.8×10^{7} molecules/dead cell[14] (instantaneous upon death);<br><br>ABs:<br>Ampicillin: $130\pm 1\ M^{-1}s^{-1}$; Benzylpenicillin $590\pm 100\ M^{-1}s^{-1}$ [50]
Single cell thresholds of antimicrobial action	AMPs:<br>$7 – 200\times 10^{6}$ molecules/per cell [reviewed in 51]

References

1. Igler, C., Rolff, J. & Regoes, R. Multi-step vs. single-step resistance evolution under different drugs, pharmacokinetics, and treatment regimens. *eLife* **10**, e64116 (2021).
2. Balaban, N. Q., Merrin, J., Chait, R., Kowalik, L. & Leibler, S. Bacterial Persistence as a Phenotypic Switch. *Science* **305**, 1622–1625 (2004).
3. Witzany, C., Regoes, R. R. & Igler, C. Assessing the relative importance of bacterial resistance, persistence and hyper-mutation for antibiotic treatment failure. *Proceedings of the Royal Society B: Biological Sciences* **289**, 20221300 (2022).
4. Windels, E. M. *et al.* Bacterial persistence promotes the evolution of antibiotic resistance by increasing survival and mutation rates. *ISME J* **13**, 1239–1251 (2019).
5. Balaban, N. Q. *et al.* Definitions and guidelines for research on antibiotic persistence. *Nat Rev Microbiol* **17**, 441–448 (2019).
6. Cao, Y., Gillespie, D. T. & Petzold, L. R. Adaptive explicit-implicit tau-leaping method with automatic tau selection. *J Chem Phys* **126**, 224101 (2007).
7. P. Johnson. Tau-Leaping Stochastic Simulation. R package version 2.2-3. (2019).
8. Brook, I. Inoculum Effect. *Reviews of Infectious Diseases* **11**, 361–368 (1989).
9. Abel zur Wiesch, P. *et al.* Classic reaction kinetics can explain complex patterns of antibiotic action. *Science Translational Medicine* **7**, 287ra73-287ra73 (2015).
10. Hedges, A. J. An examination of single-hit and multi-hit hypotheses in relation to the possible kinetics of colicin adsorption. *Journal of Theoretical Biology* **11**, 383–410 (1966).
11. Lenhard, J. R. & Bulman, Z. P. Inoculum effect of β-lactam antibiotics. *J Antimicrob Chemother* **74**, 2825–2843 (2019).

12. Wu, F. & Tan, C. Dead bacterial absorption of antimicrobial peptides underlies collective tolerance. *Journal of The Royal Society Interface* **16**, 20180701 (2019).

13. Savini, F. *et al.* Binding of an antimicrobial peptide to bacterial cells: Interaction with different species, strains and cellular components. *Biochimica et Biophysica Acta (BBA) - Biomembranes* **1862**, 183291 (2020).

14. Snoussi, M. *et al.* Heterogeneous absorption of antimicrobial peptide LL37 in Escherichia coli cells enhances population survivability. *eLife* **7**, e38174 (2018).

15. Loffredo, M. R. *et al.* Inoculum effect of antimicrobial peptides. *Proc Natl Acad Sci USA* **118**, e2014364118 (2021).

16. Levison, M. E. & Levison, J. H. Pharmacokinetics and Pharmacodynamics of Antibacterial Agents. *Infectious Disease Clinics of North America* **23**, 791–815 (2009).

17. Turfus, S. C., Delgoda, R., Picking, D. & Gurley, B. J. Chapter 25 - Pharmacokinetics. in *Pharmacognosy* (eds. Badal, S. & Delgoda, R.) 495–512 (Academic Press, 2017). doi:10.1016/B978-0-12-802104-0.00025-1.

18. Han, Y. *et al.* Multifunctional oral delivery systems for enhanced bioavailability of therapeutic peptides/proteins. *Acta Pharmaceutica Sinica B* **9**, 902–922 (2019).

19. Wagner, J. G. & Metzler, C. M. Estimation of rate constants for absorption and elimination from blood concentration data. *Journal of Pharmaceutical Sciences* **56**, 658–659 (1967).

20. Wagner, J. G. & Nelson, E. Kinetic analysis of blood levels and urinary excretion in the absorptive phase after single doses of drug. *Journal of Pharmaceutical Sciences* **53**, 1392–1403 (1964).

21. Allen, G. P., Kaatz, G. W. & Rybak, M. J. Activities of mutant prevention concentration-targeted moxifloxacin and levofloxacin against Streptococcus pneumoniae in an in

vitro pharmacodynamic model. *Antimicrobial Agents and Chemotherapy* **47**, 2606–2614 (2003).

22. Allen, G. P., Kaatz, G. W. & Rybak, M. J. In vitro activities of mutant prevention concentration-targeted concentrations of fluoroquinolones against Staphylococcus aureus in a pharmacodynamic model. *International Journal of Antimicrobial Agents* **24**, 150–160 (2004).

23. Di Grazia, A. *et al.* D-Amino acids incorporation in the frog skin-derived peptide esculentin-1a(1-21)NH2 is beneficial for its multiple functions. *Amino Acids* **47**, 2505–2519 (2015).

24. Regoes, R. R. *et al.* Pharmacodynamic Functions: a Multiparameter Approach to the Design of Antibiotic Treatment Regimens. *Antimicrobial Agents and Chemotherapy* **48**, 3670–3676 (2004).

25. Olofsson, S. K., Geli, P., Andersson, D. I. & Cars, O. Pharmacodynamic model to describe the concentration-dependent selection of cefotaxime-resistant Escherichia coli. *Antimicrobial Agents and Chemotherapy* **49**, 5081–5091 (2005).

26. Kragh, K. N. *et al.* Polymorphonuclear Leukocytes Restrict Growth of Pseudomonas aeruginosa in the Lungs of Cystic Fibrosis Patients. *Infection and Immunity* **82**, 4477–4486 (2014).

27. Yu, G., Baeder, D., Regoes, R. & Rolff, J. Predicting Drug Resistance Evolution: Antimicrobial Peptides Vs. Antibiotics. *Proc. R. Soc. Lond. B* 138107 (2018) doi:10.1101/138107.

28. Chevereau, G. *et al.* Quantifying the Determinants of Evolutionary Dynamics Leading to Drug Resistance. *PLoS Biology* **13**, 1–18 (2015).

29. Yu, G., Baeder, D. Y., Regoes, R. R. & Rolff, J. Combination Effects of Antimicrobial Peptides. *Antimicrobial Agents and Chemotherapy* **60**, 1717–1724 (2016).

30. Zahir, T. *et al.* High-throughput time-resolved morphology screening in bacteria reveals phenotypic responses to antibiotics. *Communications Biology* **2**, 1–13 (2019).

31. Thorsted, A. *et al.* Extension of pharmacokinetic/pharmacodynamic time-kill studies to include lipopolysaccharide/endotoxin release from Escherichia coli exposed to cefuroxime. *Antimicrobial Agents and Chemotherapy* **64**, 1–12 (2020).

32. Fantner, G. E., Barbero, R. J., Gray, D. S. & Belcher, A. M. Kinetics of antimicrobial peptide activity measured on individual bacterial cells using high-speed atomic force microscopy. *Nature Nanotechnology* **5**, 280–285 (2010).

33. Roversi, D. *et al.* How many antimicrobial peptide molecules kill a bacterium? The case of PMAP-23. *ACS Chemical Biology* **9**, 2003–2007 (2014).

34. Bergen, P. J., Li, J. & Nation, R. L. Dosing of colistin—back to basic PK/PD. *Current Opinion in Pharmacology* **11**, 464–469 (2011).

35. Bolintineanu, D., Hazrati, E., Davis, H. T., Lehrer, R. I. & Kaznessis, Y. N. Antimicrobial mechanism of pore-forming protegrin peptides: 100 pores to kill E. coli. *Peptides* **31**, 1–8 (2010).

36. Brogden, K. A. Antimicrobial peptides: Pore formers or metabolic inhibitors in bacteria? *Nature Reviews Microbiology* **3**, 238–250 (2005).

37. Lee, H., Popodi, E., Tang, H. & Foster, P. L. Rate and molecular spectrum of spontaneous mutations in the bacterium Escherichia coli as determined by whole-genome sequencing. *Proc Natl Acad Sci U S A* **109**, E2774-83 (2012).

38. Andersson, D. I., Hughes, D. & Kubicek-Sutherland, J. Z. Mechanisms and consequences of bacterial resistance to antimicrobial peptides. *Drug Resistance Updates* **26**, 43–57 (2016).

39. Rodríguez-Rojas, A., Makarova, O. & Rolff, J. Antimicrobials, Stress and Mutagenesis. *PLoS Pathogens* **10**, e1004445 (2014).

40. Kohanski, M. A., DePristo, M. A. & Collins, J. J. Sublethal Antibiotic Treatment Leads to Multidrug Resistance via Radical-Induced Mutagenesis. *Molecular Cell* **37**, 311–320 (2010).

41. Hall, L. M. C. & Henderson-Begg, S. K. Hypermutable bacteria isolated from humans - A critical analysis. *Microbiology* **152**, 2505–2514 (2006).

42. Pränting, M., Negrea, A., Rhen, M. & Andersson, D. I. Mechanism and fitness costs of PR-39 resistance in Salmonella enterica serovar typhimurium LT2. *Antimicrobial Agents and Chemotherapy* **52**, 2734–2741 (2008).

43. Bingen, E. *et al.* Bacterial counts in cerebrospinal fluid of children with meningitis. *Eur J Clin Microbiol Infect Dis* **9**, 278–281 (1990).

44. Feldman, W. E. Concentrations of bacteria in cerebrospinal fluid of patients with bacterial meningitis. *J Pediatr* **88**, 549–552 (1976).

45. Taylor, S. L. *et al.* Total bacterial load, inflammation, and structural lung disease in paediatric cystic fibrosis. *Journal of Cystic Fibrosis* **19**, 923–930 (2020).

46. Stressmann, F. A. *et al.* Does bacterial density in cystic fibrosis sputum increase prior to pulmonary exacerbation? *J Cyst Fibros* **10**, 357–65 (2011).

47. Carvalho, G., Guilhen, C., Balestrino, D., Forestier, C. & Mathias, J.-D. Relating switching rates between normal and persister cells to substrate and antibiotic concentrations: a mathematical modelling approach supported by experiments. *Microbial Biotechnology* **10**, 1616–1627 (2017).

48. Van den Bergh, B. *et al.* Frequency of antibiotic application drives rapid evolutionary adaptation of Escherichia coli persistence. *Nat Microbiol* **1**, 1–7 (2016).

49. Rodríguez-Rojas, A., Baeder, D. Y., Johnston, P., Regoes, R. R. & Rolff, J. Bacteria primed by antimicrobial peptides develop tolerance and persist. *PLOS Pathogens* **17**, e1009443 (2021).

50. Abel zur Wiesch, P. *et al.* Classic reaction kinetics can explain complex patterns of antibiotic action. *Sci. Transl. Med.* **7**, (2015).

51. Savini, F., Bobone, S., Roversi, D., Mangoni, M. L. & Stella, L. From liposomes to cells: Filling the gap between physicochemical and microbiological studies of the activity and selectivity of host-defense peptides. *Peptide Science* **110**, e24041 (2018).
